# Efficient and Exact Sampling of Simple Graphs with Given Arbitrary Degree Sequence

**DOI:** 10.1371/journal.pone.0010012

**Published:** 2010-04-08

**Authors:** Charo I. Del Genio, Hyunju Kim, Zoltán Toroczkai, Kevin E. Bassler

**Affiliations:** 1 Department of Physics, University of Houston, Houston, Texas, United States of America; 2 Texas Center for Superconductivity (TcSUH), University of Houston, Houston, Texas, United States of America; 3 Interdisciplinary Center for Network Science and Applications (iCeNSA), University of Notre Dame, Notre Dame, Indiana, United States of America; University of East Piedmont, Italy

## Abstract

Uniform sampling from graphical realizations of a given degree sequence is a fundamental component in simulation-based measurements of network observables, with applications ranging from epidemics, through social networks to Internet modeling. Existing graph sampling methods are either link-swap based (Markov-Chain Monte Carlo algorithms) or stub-matching based (the Configuration Model). Both types are ill-controlled, with typically unknown mixing times for link-swap methods and uncontrolled rejections for the Configuration Model. Here we propose an efficient, polynomial time algorithm that generates statistically independent graph samples with a given, arbitrary, degree sequence. The algorithm provides a weight associated with each sample, allowing the observable to be measured either uniformly over the graph ensemble, or, alternatively, with a desired distribution. Unlike other algorithms, this method always produces a sample, without back-tracking or rejections. Using a central limit theorem-based reasoning, we argue, that for large 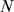, and for degree sequences admitting many realizations, the sample weights are expected to have a lognormal distribution. As examples, we apply our algorithm to generate networks with degree sequences drawn from power-law distributions and from binomial distributions.

## Introduction

Network representation has become an increasingly widespread methodology of analysis to gain insight into the behavior of complex systems, ranging from gene regulatory networks to human infrastructures such as the Internet, power-grids and airline transportation, through metabolism, epidemics and social sciences [1]–[4]. These studies are primarily data driven, where connectivity information is collected, and the structural properties of the resulting graphs are analyzed for modeling purposes. However, rather frequently, full connectivity data is unavailable, and the modeling has to resort to considerations on the *class of graphs* that obeys the available structural data. A rather typical situation is when the only information available about the network is the degree sequence of its nodes 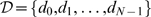. For example, in epidemiology studies of sexually transmitted diseases [5], anonymous surveys may only collect the *number* of sexual partners of a person in a given period of time, not their identity. Epidemiologists are then faced with constructing a *typical* contact graph having the observed degree sequence, on which disease spread scenarios can be tested. Another reason for studying classes or *ensembles* of graphs obeying constraints comes from the fact that the network structure of many large-scale real-world systems is not the result of a global design, but of complex dynamical processes with many stochastic elements. Accordingly, a statistical mechanics approach [1] can be employed to characterize the collective properties of the system emerging from its node level (microscopic) properties. In this approach, statistical ensembles of graphs are defined [6], [7], representing “connectivity microstates” from which macroscopic system level properties are inferred via averaging. Here we focus on the degree as a node characteristic, which could represent, for example, the number of friends of a person, the valence of an atom in a chemical compound, the number of clients of a router, etc.

In spite of its practical importance, finding a method to construct degree-based graphs in a way that allows the corresponding graph ensemble to be properly sampled has been a long-standing open problem in the network modeling community (references using various approaches are given below). Here we present a solution to this problem, using a biased sampling approach. We consider degree-based graph ensembles on two levels: 1) sequence-level, where a specific sequence of degrees is given, and 2) distribution level, where the sequences are themselves drawn from a given degree distribution 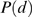. In the remainder we will focus on the fundamental case of labeled, undirected simple graphs. In a simple graph any link connects a single pair of distinct nodes and self loops and multiple links between the same pair of nodes are not allowed. Without loss of generality, consider a sequence of 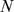 positive integers 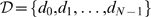, arranged in non-increasing order: 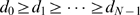. If there is at least one simple graph 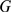 with degree sequence 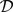, the sequence 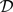 is called a *graphical sequence* and we say that 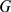 *realizes* 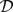. Note that not every sequence of positive integers can be realized by simple graphs. For example, there is no simple graph with degree sequence 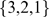 or 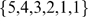, while the sequence 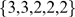 can obviously be realized by a simple graph. In general, if a sequence is graphical, then there can be several graphs having the same degree sequence. Also note that given a graphical sequence, the careless or random placing of links between the nodes may not result in a simple graph.

Recently, a direct, swap-free method to systematically construct all the simple graphs realizing a given graphical sequence 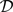 was presented [8]. However, in general (for exceptions see Ref. [9]), the number of elements of the set 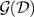 of all graphs that realize sequence 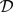, increases very quickly with 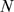: a simple upper bound is provided by the number of all graphs with sequence 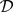, allowing for multiple links and loops: 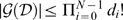. Thus, typically, systematically constructing all graphs with a given sequence 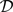 is practical only for short sequences, such as when determining the structural isomers of alkanes [8]. For larger sequences, and in particular for modeling real-world complex networks, it becomes necessary to sample 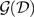. Accordingly, several variants based on the Markov Chain Monte Carlo (MCMC) method were developed. They use link-swaps [10] (“switches”) to produce pseudo-random samples from 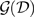. Unfortunately, most of them are based on heuristics, and apart from some special sequences, little has been rigorously shown about the methods' mixing time, and accordingly they are ill-controlled. The literature on such MCMC methods is simply too extensive to be reviewed here, instead, we refer the interested reader to Refs. [11]–[13] and the references therein. Finally, we recall the main swap-free method producing uniform random samples from 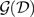, namely the configuration model (CM) [14]–[17]. This method picks a pair of nodes uniformly at random and connects them, until a rejection occurs due to a double link or a self-loop, in which case it restarts from the very beginning. For this reason, the CM can become very slow, as shown in the Discussion section. The CM has inspired approximation methods as well [18] and methods that construct random graphs with given *expected* degrees [19].

Here, by developing new results from the theorems in Ref. [8], we present an efficient algorithm that solves this fundamental graph sampling problem, and it is exact in the sense that it is not based on any heuristics. Given a graphical sequence, the algorithm always finishes with a simple graph realization in polynomial time, and it is rejection free. While the samples obtained are not uniformly generated, the algorithm also provides the exact weight for each sample, which can then be used to produce averages of arbitrary graph observables measured uniformly, or following any given distribution over 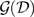.

## Methods

### Mathematical foundations

Before introducing the algorithm, we state some results that will be useful later on. We begin with the Erdös-Gallai (EG) theorem [20], which is a fundamental result that allows us to determine whether a given sequence of non-negative integers, called “degree sequence” hereafter, is graphical.

#### Theorem 1 (Erdö-Gallai)

*A non-increasing degree sequence* 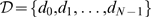 *is graphical if and only if their sum is even and, for all* 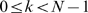:
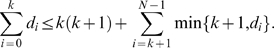
(1)


A necessary and sufficient condition for the graphicality of a degree sequence, which is constrained from having links between some node and a “forbidden set” of other nodes is given by the star-constrained graphicality theorem [8]. In this case the forbidden links are all incident on one node and thus form a “star”. To state the theorem, we first define the “leftmost adjacency set” of a node 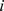 with degree 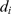 in a degree sequence 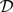 as the set consisting of the 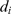 nodes with the largest degrees that are *not in* the forbidden set. If 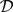 is non-increasing, then the nodes in the leftmost adjacency set are the first 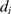 nodes in the sequence that are not in the forbidden set. The forbidden set could represent nodes that are either already connected to 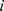, and thus subsequent connections to them are forbidden, or just imposed arbitrarily. Using this definition, the theorem is:

#### Theorem 2 (Star-constrained graphical sequences)

*Let* 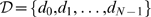 *be a non-increasing graphical degree sequence*. *Assume there is a set of forbidden links incident on a node* 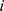. *Then a simple graph avoiding the forbidden links can be constructed if and only if a simple graph can be constructed where* 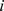 *is connected to all the nodes in its leftmost adjacency set*.

A direct consequence [8] of Theorem 2 for the case of an empty forbidden set is the well-known Havel-Hakimi result [21], [22], which in turn implies:

#### Corollary 1

*Let* 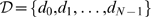 *be a non-increasing unconstrained graphical degree sequence*. *Then*, *given any node* 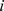, *there is a realization of* 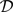 *that includes a link between the first node and* 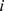.

Another result we exploit here is Lemma 3 of Ref. [8], extended to star-constrained sequences:

#### Lemma 1

*Let* 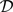 *be a graphical sequence, possibly with a star constraint incident on node* 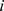. *Let* 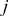 *and* 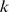 *be distinct nodes not in the forbidden set and different from* 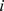, *such that* 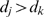. *Then* 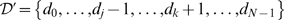 *is also a graphical sequence with the same star constraint*.

*Proof.* Let 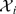 denote the set of nodes forbidden to connect to node 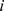. Since 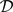 is star-constrained graphical there is a simple graph 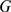 realizing the sequence with no connections between 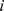 and 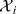. Since 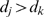, there is a node 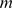 to which 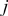 is connected but 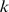 is not. Note that 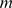 could be in 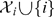. Now cut the edge 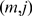 of 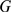 creating a stub at 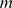 and another at 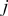. Remove the stub at 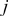 so that its degree becomes 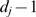, and add a stub at 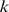 so that its degree becoming 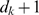. Since there are no connections in 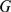 between 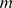 and 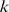, connect the two stubs at these nodes creating a simple graph 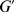 thus realizing 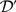. Clearly there are still no connections between 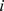 and 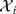 in 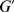, and thus 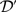 is also star-constrained graphical.

Finally, using Lemma 1 and Theorem 2, we prove:

#### Theorem 3

*Let* 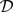 *be a degree sequence*, *possibly with a star-constraint incident on node* 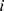, *and let* 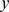 *and* 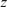 *be two nodes with degrees such that* 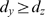 *that are not constrained from linking to node* 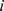. *If the residual degree sequence* 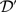 *obtained from* 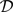 *by reducing the degrees at* 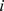 *and* 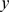 *by unity is not graphical*, *then the degree sequence* 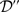 *obtained from* 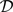 *by reducing the degrees at* 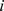 *and* 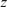 *by unity is also not graphical*.

*Proof.* By definition, 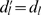 for 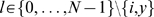 and 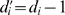, 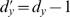; 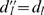 for 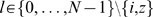 and 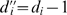, 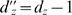. We consider 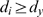, however, the proof is not affected by this assumption. By assumption, 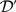 is not graphical. Using proof by contradiction, assume that 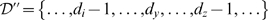 is graphical. Clearly, 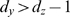, and thus we can apply Lemma 1 on this sequence. As a result, the sequence 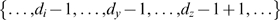, that is exactly 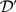 is graphical, a contradiction.

Note that if a sequence is non-graphical, then it is not star-constrained graphical either, and thus Theorem 3 is in its strongest form.

### Biased sampling

The sampling algorithm described below is ergodic in the sense that every possible simple graph with the given finite degree sequence is generated with non-zero probability. However, it does not generate the samples with uniform probability; the sampling is biased. Nevertheless, the algorithm can be used to compute network observables that are unbiased, by appropriately weighing the averages measured from the samples. According to a well known principle of biased sampling [23],[24], if the relative probability of generating a particular sample 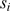 is 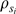, then an unbiased estimator for an observable 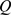 measured from a set of 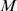 randomly generated samples 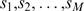 is the weighted average
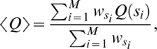
(2)where the weights are 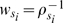, and the denominator is a normalization factor. The key to this method is to find the appropriate weight 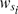 to associate with each sample. Note that in addition to uniform sampling, it is in fact possible to sample with any arbitrary distribution by choosing an appropriate set of sample weights.

## Results

### The algorithm

Let 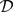 be a non-increasing graphical sequence. We wish to sample the set 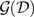 of graphs that realize this sequence. The graphs can be systematically constructed by forming all the links involving each node. To do so, begin by choosing the first node in the sequence as the “hub” node and then build the set of the “allowed nodes” 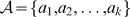 that can be connected to it. 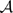 contains all the nodes that can be connected to the hub such that if a link is placed between the hub and a node from 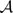, then a simple graph can still be constructed, thus preserving graphicality. Choose uniformly at random a node 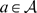, and place a link between 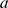 and the hub. If 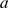 still has “stubs”, i.e. remaining links to be placed, then add it to the set of “forbidden nodes” 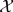 that contains all the nodes which can't be linked anymore to the hub node and which initially contains only the hub; otherwise, if 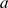 has no more stubs to connect, then remove it from further consideration. Repeat the construction of 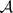 and link the hub with one of its randomly chosen elements until the stubs of the hub are exhausted. Then remove the hub from further consideration, and repeat the whole procedure until all the links are made and the sample construction is complete. Each time the procedure is repeated, the degree sequence 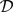 considered is the “residual degree sequence”, that is the original degree sequence reduced by the links that have previously been made, and with any zero residual degree node removed from the sequence. Then, choose a new hub, empty the set of forbidden nodes 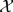 and add the new hub to it. It is convenient, but not necessary, to choose the new hub to be a node with maximum degree in the residual degree sequence.

The sample weights needed to obtain unbiased estimates using Eq. 2 are the inverse relative probabilities of generating the particular samples. If in the course of the construction of the sample 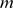 different nodes 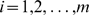 are chosen as the hub and they have 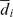 residual degrees when they are chosen, then this sample weight can be computed by first taking the product of the sizes 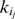 of the allowed sets 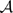 constructed, then dividing this quantity by a combinatorial factor which is the product of the factorials of the residual degrees of each hub:
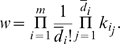
(3)The weight accounts for the fact that at each step the hub node has 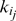 nodes it can be linked to, which is the size of the allowed set at that point, and that the number of equivalent ways to connect the residual stubs of a new hub is 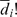. Note that it is always true that 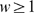, with 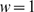 occurring for sequences for which there is only one possible graph.

#### Building the allowed set

The most difficult step in the sampling algorithm is to construct the set of allowed nodes 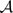. In order to do so first note that Theorem 3 implies that if a non-forbidden node, that is a node not in 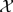, can be added to 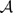, then all non-forbidden nodes with equal or higher degree can also be added to 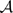. Conversely, if it is determined that a non-forbidden node cannot be added to 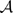, then all nodes with equal or smaller degree also cannot be added to 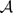. Therefore, referring to the degrees of nodes that cannot be added to 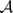 as “fail-degrees”, the key to efficiently construct 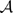 is to determine the maximum fail-degree, if fail-degrees exist.

The first time 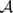 is constructed for a new hub, according to Corollary 1, there is no fail-degree and 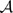 consists of all the other nodes. However, constructing 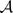 becomes more difficult once links have been placed from the hub to other nodes. In this case, to find the maximum fail-degree note that at any step during the construction of a sample the residual sequence being used is graphical. Then, since according to Theorem 2 any connection to the leftmost adjacency set of the hub preserves graphicality, it follows from Theorem 3 that any fail-degree has to be strictly less than the degree of any node in the leftmost adjacency set of the hub.

If there are non-forbidden nodes in the residual degree sequence that have degree less than any in its leftmost adjacency set, then the maximum fail-degree can be found with a procedure that exploits Theorem 2. In particular, if the hub is connected to a node with a fail-degree, then, by Theorem 2, even if all the remaining links from the hub were connected to the remaining nodes in the leftmost adjacency set, the residual sequence will not be graphical. Our method to find fail-degrees, given below, is based on this argument.

Begin by constructing a new residual sequence 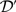 by temporarily assuming that links exist between the hub and all the nodes in its leftmost adjacency set *except for the last one*, which has the lowest degree in the set. The nodes temporarily linked to the hub should also be temporarily added to the set of forbidden nodes 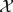. The nodes in 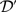 should be ordered so that it is non-increasing, that forbidden nodes appear before non-forbidden nodes of the same degree, and that the hub, which now has residual degree 1, is last.

At this point, in principle one could find the maximum fail degree by systematically connecting the last link of the hub with non-forbidden nodes of decreasing degree, and testing each time for graphicality using Theorem 1. If it is not graphical then the degree of the last node connected to the hub is a fail-degree, and the node with the largest degree for which this is true will have the maximum fail-degree. However, this procedure is inefficient because each time a new node is linked with the hub the residual sequence changes and every new sequence must be tested for graphicality.

A more efficient procedure to find the maximum fail-degree instead involves only testing the sequence 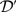. To see how this can be done, note that 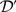 is a graphical sequence, by Theorem 2. Thus, by Theorem 1, for all relevant values of 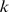, the left hand side of Inequality 1, 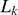, and the right hand side of it, 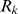, satisfy 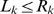. Furthermore, for the purposes of finding fail-degrees it is sufficient to consider linking the final stub of the hub with only the last non-forbidden node of a given degree, if any exists. After any such link is made, the resulting degree-sequence 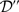 will be non-increasing, and thus Theorem 1 can be applied to test it for graphicality. Therefore, if the degree of the node connected with the last stub of the hub is a fail-degree, then Inequality 1 for 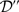 must fail for some 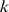. For each 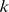, the possible differences in 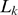 and 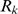 between 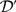 and 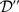 are as follows. 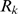 is always reduced by 1 because the residual degree of the hub is reduced from 1 to 0. 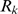 may be reduced by an another factor of 1 if the last node connected to the hub, having index 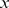 and degree 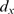, is such that 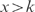 and 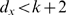. 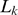 is reduced by 1 if 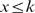, otherwise it is unchanged.

Considering these conditions that can cause Inequality 1 to fail for 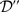, the set of allowed nodes 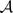 can be constructed with the following algorithm that requires only testing 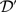. Starting with 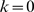, compute the values of 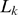 and 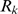 for 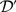. There are three possible cases: (1) 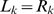, (2) 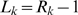, and (3) 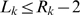. In case (1) fail-degrees occur whenever 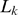 is unchanged by making the final link to the hub. Thus, the degree of the first non-forbidden node whose index is greater than 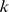 is the largest fail-degree found with this value of 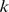. In case (2) fail-degrees occur whenever 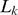 is unchanged and 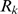 is reduced by 2 by making the final link to the hub. Thus, the degree of the first non-forbidden node whose index is greater than 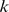 and whose degree is less than 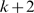 is the largest fail-degree found with this value of 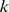. In case (3) no fail-degree can be found with this value of 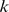. Repeat this process sequentially increasing 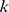, until all the relevant 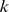 values have been considered, then retain the maximum fail-degree. It can be shown that the algorithm can be stopped either after a case (1) occurs, or after 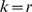 where 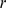 is the lowest index of any node in 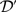 with degree 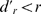. Once the maximum fail-degree is found, remove the nodes that were temporarily added to 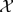 and construct 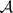 by including all non-forbidden nodes of 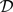 with a higher degree. If no fail-degree is ever found, then all non-forbidden nodes of 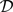 are included in 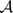. 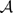 will always include the leftmost adjacency set of the hub and any non-forbidden nodes of equal degree.

Note that after a link is placed in the sample construction process, the residual degree sequence 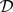 changes, and therefore, 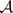 has to be determined every time.

#### Implementing the Erdös-Gallai test

Finally, 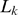 and 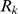 should be calculated efficiently. Calculating the sums that comprise them for each new value of 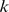 can be computationally intensive, especially for long sequences. Even computing them only for as many distinct terms as there are in the sequence, as suggested in Ref. [25], can still become slow if the degree distribution is not quickly decreasing. Instead, it is much more efficient to use recurrence relations to calculate them.

A recurrence relation for 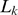 is simply
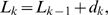
(4)with 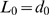.

For non-increasing degree sequences, define the “crossing-index” 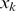 for each 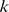 as the index of first node that has degree less than 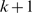, that is for which 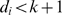 for all 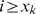. If no such index exists, such as for 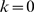 since the minimum degree of any node in the sequence is 1, then set 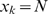. Then, a recurrence relation for 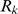 is
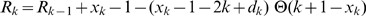
(5)where 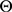 is a discrete equivalent of the Heaviside function, defined to be 1 on positive integers and 0 otherwise, and 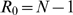. Or, since the crossing-index can not increase with 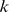, that is 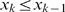 for all 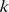, a value 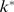 will exist for which 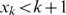 for all 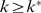, and so Eq. 5 can be written
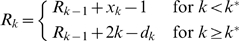
(6)Thus, there is no need to find 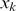 for 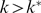.

Using Eqs. 4 and 6, the mechanism of the calculation of 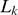 and 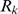 at sequential values of 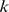 is shifted from a slow repeated calculation of sums of many terms to the much less computationally intensive task of calculating the recurrence relations. In order to perform the test efficiently, a table of the values of crossing-index 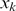 for each relevant 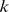 can be created as 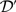 is constructed.

It should be noted that the usefulness of this method for calculating 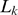 and 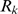 is broader than its use for calculating fail-degrees in our sampling algorithm. In particular, it can be used in an Erdös-Gallai test to efficiently determine whether a degree-sequence is graphical.

### Sample weights

As previously stated, the weight 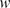 associated with a particular sample, given by Eq. 3, is the product of the sizes 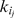 of all the sets of allowed nodes that have been built for each hub node 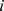 divided by the product of the factorials of the initial residual degrees of each hub node. The logarithm of this weight is
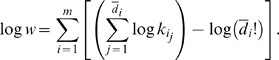
(7)Generally, degree sequences with 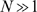 admit many graphical realizations. When this is true, each of the 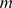 terms in square brackets in Eq. 7 are effectively random and independent, and, by virtue of the central limit theorem, their sum will be normally distributed. That is, the weight 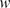 of graph samples generated from a given degree sequence with large 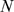 is typically log-normally distributed. However, degree sequences with 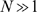 that have only a small number of realizations do exist, and 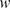 is not expected to be log-normally distributed for those sequences.

**Figure 1:**
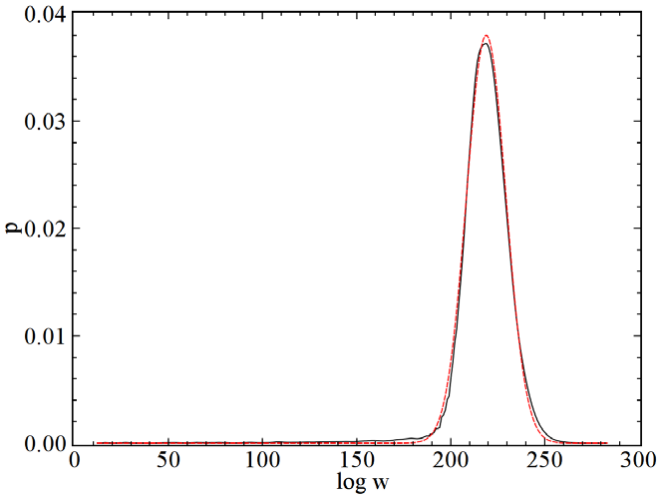
Probability distribution 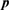 of the logarithm of weights for an ensemble of power-law sequences with 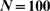 and 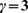. The ensemble contained 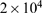 graphical sequences, and for each sequence 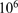 graph samples were produced. Thus, the total number of samples produced was 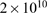. The simulation data is given by the solid black line and a Gaussian fit of the data is shown by the dashed red line that nearly obscures the black line.

Furthermore, one can consider not just samples of a particular graphical sequence, but of an ensemble of sequences. By a similar argument to that given above for individual sequences, the weight 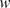 of graph samples generated from an ensemble of sequences will also typically be log-normally distributed in the limit of large 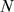. For example, consider an ensemble of sequences of randomly chosen power-law distributed degrees, that is, sequences of random integers chosen from a probability distribution 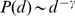. Hereafter, we refer to such sequences as “power-law sequences.” Figure 1 shows the probability distribution of the logarithm of weights for realizations of power-law sequences with exponent 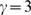 and 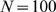. Note that this distribution is well approximated by a Gaussian fit.

**Figure 2:**
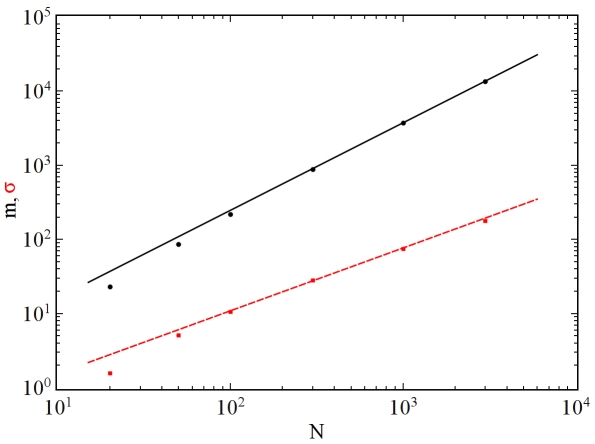
Mean 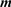 and standard deviation 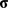 of the distributions of the logarithm of the weights vs. number of nodes 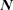 of samples from an ensemble of power-law sequences with 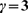. The black circles correspond to 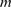, the red squares correspond to 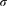. The error bars are smaller than the symbols. The solid black line and the dashed red line show the outcomes of fits on the data. The linearity of the data on a logarithmic scale indicates that the 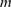 and 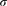 follow power-law scaling relations with 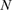: 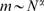 and 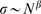. The slopes of the fit lines are an estimate of the value of the exponents: 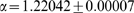 and 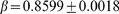.

We have also studied the behavior of the mean and the standard deviation of the probability distribution of the logarithm of the weights of such power-law sequences as a function of 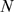. As shown in Fig. 2, they scale as a power-law. We have found qualitatively similar results, including power-law scaling of the growth of the mean and variance of the distribution of 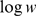, for binomially distributed degree sequences that correspond to those of Erdös-Renyi random graphs with node connection probability 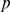 such that 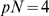, and for uniformly distributed degree sequences, that is power-law sequences with 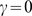, with an upper limit, or cutoff, of 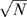 for the degree of a node. However, for uniformly distributed degree sequences without an imposed upper limit on node degrees, we find that the sample weights are not log-normally distributed.

### Complexity

In this section we discuss the algorithm's computational complexity. We first provide an upper bound on the worst case complexity, given a degree sequence 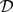. Then, using extreme value arguments, we conservatively estimate the average case complexity for degree sequences of random integers chosen from a distribution 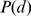. The latter is useful for realistically estimating the computational costs for sampling graphs from ensembles of long sequences.

To determine an upper bound on the worst case complexity for constructing a sample from a given degree sequence 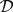, recall that the algorithm connects all the stubs of the current hub node before it moves on to the hub node of the new residual sequence. For every stub from the hub one must construct the allowed set 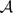. The algorithm for constructing 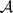, which includes constructing 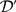, performing the 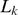 vs 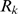 comparisons, and determining the maximum fail-degree, can be completed in 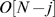 steps, where 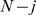 is the maximum possible number of nodes in the residual sequence after eliminating 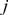 hubs from the process. Therefore, an upper bound on the worst case complexity 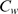 of the algorithm given a sequence 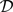 is:
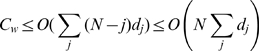
(8)where the sum involves at most 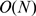 terms. Equivalently, 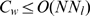, with 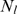 being the number of links in the graph. For simple graphs, the maximum possible number of links is 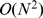, and the minimum possible number is 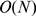. If 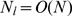, then 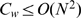, and if 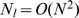, then 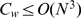, which is an upper bound, independent of the sequence.

From Eq. 8, the expected complexity for the algorithm to construct a sample for a degree sequence of random integers chosen from a distribution 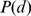, normalized to unity, can be conservatively estimated as
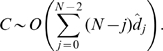
(9)Here 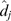 is the expectation value for the degree of the node with index 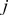, which is the largest degree for which the expected number of nodes with equal or larger degree is at least 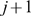. That is,
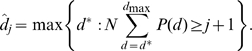
(10)Notice that the sum in the above equation runs to the maximum allowed degree in the network 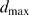, which is nominally 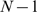, but a different value can be imposed. For example, in the case of power-law sequences, the so-called structural cutoff of 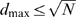 is necessary if degree correlations are to be avoided [19], [26], [27]. However, such a cutoff needs to be imposed only for 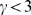, because the expected maximum degree 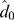 in a power-law network grows like 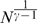. Thus, for 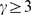, 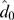 grows no faster than 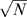 and no degree correlations exist for large 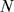 [28].

**Figure 3:**
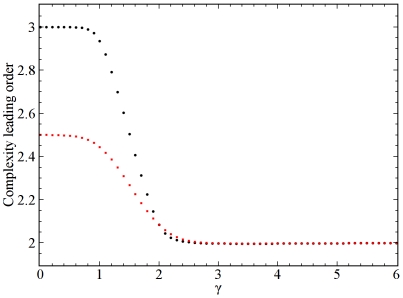
The estimated computational complexity of the algorithm for power-law sequences. The leading order of the computational complexity of the algorithm as a power of 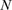, where 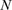 is the number of nodes, is plotted as a function of the degree distribution power-law exponent 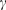. The black circles correspond to ensembles of sequences without cutoff, while the red squares correspond to ensembles of sequences with structural cutoff in the maximum degree of 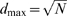. The fits that yielded the data points were carried out considering sequences ranging in size from 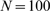 to 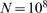.

Given a particular form of distribution 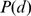, Eq. 9 can be computed for different values of 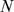. Subsequent fits of the results to a power-law function allow the order of the complexity of the algorithm to be estimated. Figure 3 shows the results of such calculations for power-law sequences with and without the structural cutoff of 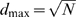 as a function of exponent 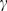. Note that, in the absence of cutoff, the results indicate that the order of the complexity goes to a value of 3 for 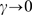, that is, in the limit of a uniform degree distribution. However, if the structural cutoff is imposed the order of the complexity is only 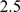 in this limit. Both these results are easily verified analytically.

We have tested the estimates shown in Fig. 3 with our implementation of the sampling algorithm for power-law sequences with and without the structural cutoff for certain values of 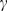, including 0, 2, and 3. This was done by measuring the actual execution times for generating samples for different 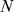 and fitting the results to a power-law function. In every case, the actual order of the complexity of our implementation of the sampling algorithm was equal to or slightly less than its estimated value shown in Fig. 3.

## Discussion

We have solved the long standing problem of how to efficiently and accurately sample the possible graphs of any graphical degree sequence, and of any ensemble of degree sequences. The algorithm we present for this purpose is ergodic and is guaranteed to produce an independent sample in, at most, 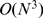 steps. Although the algorithm generates samples non-uniformly, and, thus, it is biased, the relative probability of generating each sample can be calculated explicitly permitting unbiased measurements to be made. Furthermore, because the sample weights are known explicitly, the algorithm makes it possible to sample with any arbitrary distribution by appropriate re-weighting.

It is important to note that the sampling algorithm is guaranteed to successfully and systematically proceed in constructing a graph. This behavior contrasts with that of other algorithms, such as the configuration model (CM), which can run into dead ends that require back-tracking or restarting, leading to considerable losses of time and potentially introducing an uncontrollable bias into the results. While there are classes of sequences for which it is perhaps preferable to use the CM instead of our algorithm, in other cases its performance relative to ours can be remarkably poor. For example, a configuration model code failed to produce even a single sample of a uniformly distributed graphical sequence, 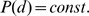, with 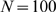, after running for more than 24 hours, while our algorithm produced 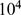 samples of the very same sequence in 30 seconds. Furthermore, each sample generated by our algorithm is independent. This behavior contrasts with that of algorithms based on MCMC methods. Because our algorithm works for any graphical sequence and for any ensemble of random sequences, it allows arbitrary classes of graphs to be studied.

One of the features of our algorithm that makes it efficient is a method of calculating the left and right sides of the inequality in the Erdös-Gallai theorem using recursion relations. Testing a sequence for graphicality can thus be accomplished without requiring repeated computations of long sums, and the method is efficient even when the sequence is nearly non-degenerate. The usefulness of this method is not limited to the algorithm presented for graph sampling, but can be used anytime a fast test of the graphicality of a sequence of integers is needed.

There are now over 6000 publications focusing on complex networks. In many of these publications various processes, such as network growth, flow on networks, epidemics, etc., are studied on toy network models used as “graph representatives” simply because they have become customary to study processes on. These include the Erdös-Rényi random graph model, the Barabási-Albert preferential attachment model, the Watts-Strogatz small-world network model, random geometric graphs, etc. However, these toy models are based on specific processes that constrain their structure beyond their degree-distribution, which in turn might not actually correspond to the processes that have led to the structure of the networks investigated with them, thus potentially introducing dangerous biases in the conclusions of these studies. The algorithm presented here provides a way to study classes of simple graphs constrained solely by their degree sequence, and nothing else. However, additional constraints, such as connectedness, or any functional of the adjacency matrix of the graph being constructed, can in principle be added to the algorithm to further restrict the graph class built.

After this paper was accepted for publication, we became aware of an unpublished work by J. Blitzstein and P. Diaconis that provides another direct construction method for sampling graphs with given degree sequences.
